# Surgical repair of partial anomalous pulmonary venous return with intact atrial septum in a 65-year-old woman: a case report

**DOI:** 10.1186/s13256-018-1874-x

**Published:** 2018-11-24

**Authors:** Narihiro Ishida, Katsuya Shimabukuro, Shojiro Yamaguchi, Etsuji Umeda, Hiroki Ogura, Shohei Mitta, Ryutaro Kimata, Hirofumi Takemura, Kiyoshi Doi

**Affiliations:** 1 Department of General and Cardiothoracic Surgery, Graduate School of Medicine, Gifu University, 1-1 Yanagido, Gifu City, 501-1194 Japan; 2 Department of Thoracic, Cardiovascular and General Surgery, Kanazawa University, Kanazawa City, 920-8641 Japan

**Keywords:** Congenital heart disease, Older adult, Cardiac pathology, Pulmonary vein

## Abstract

**Background:**

Partial anomalous pulmonary venous return is a rare congenital cardiac anomaly that usually involves the right pulmonary vein and an atrial septal defect. Isolated partial anomalous pulmonary venous return with an intact atrial septum is even rarer, and this condition is usually treated surgically in younger patients. We describe isolated partial anomalous pulmonary venous return in a 65-year-old woman who was treated by caval division with pericardial patch baffling through a surgically created atrial septal defect and reconstruction of the superior vena cava using a prosthetic graft.

**Case presentation:**

A 65-year-old Asian woman who presented with exertional dyspnea was diagnosed with isolated partial anomalous pulmonary venous return. The surgical indications and strategy were controversial because of the rarity of this pathology. She had an indication for surgery because she was symptomatic and had a high ratio of pulmonary to systemic blood flow. We considered that surgical procedures should avoid postoperative stenosis of a reconstructed flow tract, sinus node dysfunction, and thrombogenesis. We created a caval division with pericardial patch baffling through a surgically created atrial septal defect and reconstructed the superior vena cava using a prosthetic graft for the isolated partial anomalous pulmonary venous return. She has since remained free of exertional dyspnea, arrhythmia, and thrombotic complications. This surgical strategy is safe and effective for treating isolated partial anomalous pulmonary venous return in older symptomatic adults.

**Conclusions:**

The long-term outcome of surgical repair of partial anomalous pulmonary venous return with an intact atrial septum in our patient, a symptomatic 65-year-old woman, was excellent.

## Background

Partial anomalous pulmonary venous return (PAPVR) is a rare congenital heart disease that usually involves the right pulmonary vein (PV) and an atrial septal defect (ASD). Isolated PAPVR with an intact atrial septum is even rarer; thus, surgical indications and strategies remain controversial. Single- and double-patch techniques are effective for treating PAPVR. However, concerns have been raised regarding postoperative stenosis or obstruction of the reconstructed flow tract and sinus node (SN) dysfunction, particularly in PAPVR when the right upper pulmonary vein (RUPV) inflows into the upper superior vena cava (SVC). Few surgical reports have described an older adult with PAPVR accompanied by an intact atrial septum. We describe PAPVR repair using caval division, a surgically created ASD, and prosthetic reconstruction.

## Case presentation

**Fig. 1:**
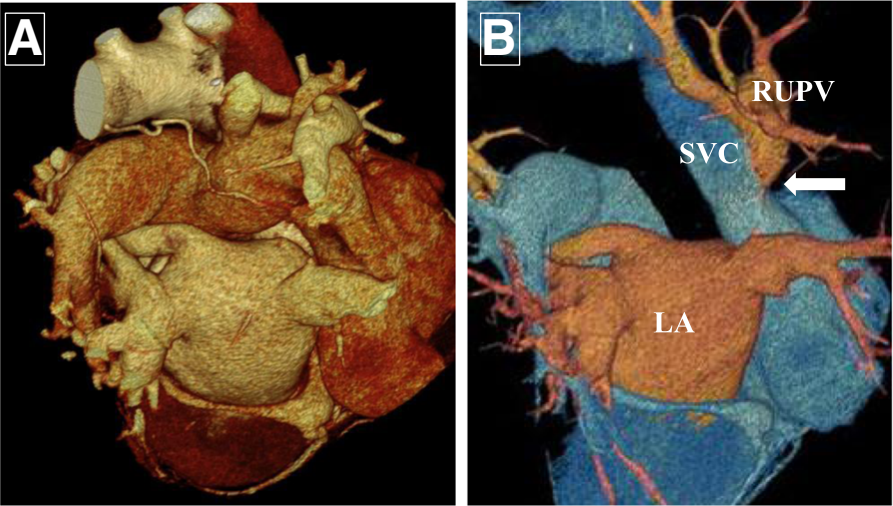
Preoperative three-dimensional computed tomography findings. **a** Dorsal view. **b** Image excluding right pulmonary artery. *Arrow* indicates where right upper pulmonary vein (RUPV) connects to the superior vena cava (SVC) rather than the left atrium (LA)

A 65-year-old Asian woman was admitted with exertional dyspnea. She had a medical history of hypertension, hyperlipidemia, and coronary artery disease that had required transcatheter intervention. Her family history also included coronary artery disease. Her social history was unremarkable with respect to environmental and workplace exposures, and she did not smoke or consume alcohol. A physical examination upon admission revealed no signs of cardiac congestion or neurological deficits. Her vital signs were a systemic blood pressure of 143/86 mmHg, pulse rate of 89 beats per minute, and a body temperature of 35.9 °C. Chest x-rays at the time of admission showed cardiomegaly with protrusion of the right first aortic arch, and electrocardiography showed an incomplete right bundle branch block of normal sinus rhythm. Coronary angiography performed because of her history of coronary artery disease revealed no significant coronary arterial stenosis. However, a concurrent pressure study revealed oxygen step-up in gas sampling between the SVC and right atrium (RA) with a pulmonary blood flow/systemic blood flow ratio (Qp/Qs) of 2.07, even though pulmonary artery pressure was normal in the pressure study and the atrial septum was intact on echocardiography. Contrast-enhanced computed tomography (CT) revealed that the RUPV flowed into the SVC (Fig. 1) without any other congenital heart conditions, including an ASD and a dilated RA and right ventricle (RV). Isolated PAPVR was diagnosed on the basis of CT imaging findings, and surgery was indicated on the basis of the patient’s symptoms and the hemodynamic pressure findings.

**Fig. 2:**
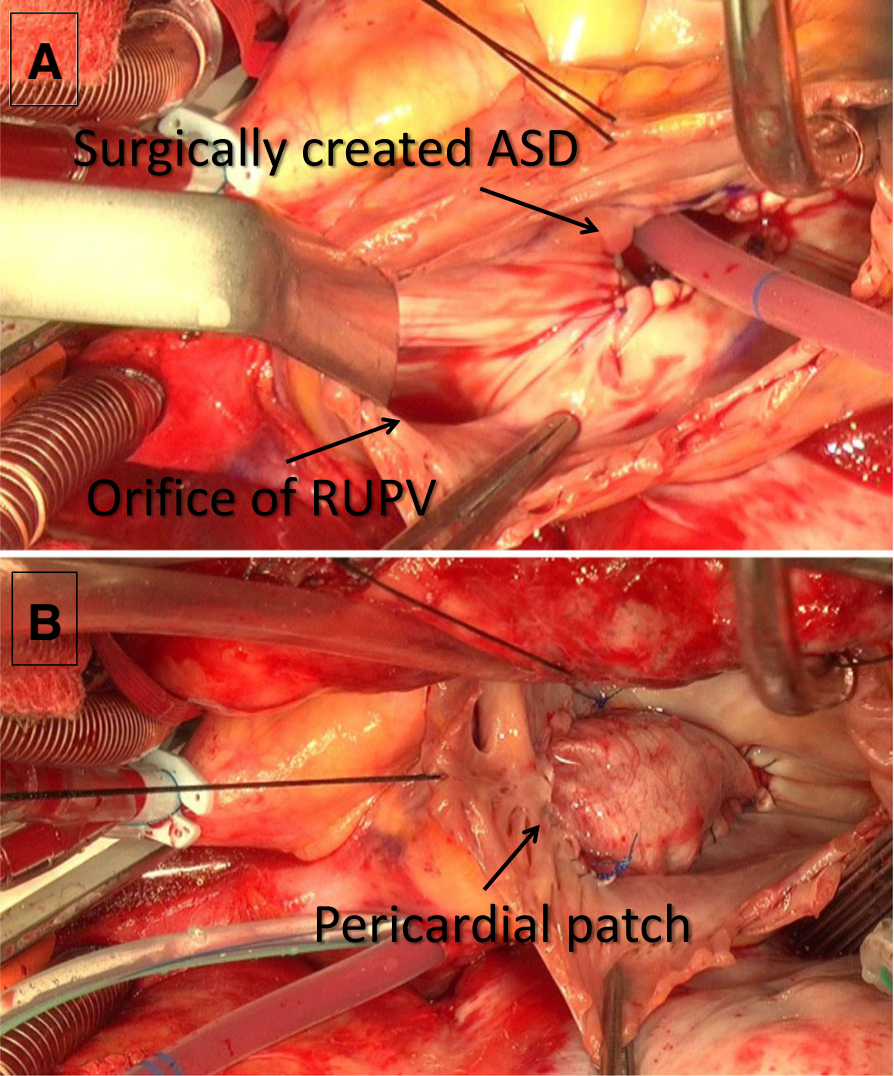
Intraoperative imaging findings. **a** Surgically created atrial septal defect (ASD) and right upper pulmonary vein (RUPV) orifice. **b** Atrial division with autologous pericardial patch baffle

The surgical approach was via a median sternotomy. The SVC was cannulated near the confluence of the innominate vein, sufficiently above the RUPV. A cardiopulmonary bypass was established, and cardiac arrest was applied. The RA was incised longitudinally. The intact atrial septum was incised in the cranial direction from the superior aspect of the fossa ovalis, and this new ASD formed a hole with an approximate diameter of 15 mm after the right and left atrial endocardia were closed using continuous 5-0 polypropylene sutures (Fig. 2a). An intra-atrial baffle comprising an autologous pericardial patch was sutured from the inferior border of the new ASD to the cavoatrial junction to separate the cavity between the RA and the SVC (Fig. 2b). The SVC was divided above the confluence of the RUPV, and the cardiac side of the SVC stump was sutured closed. A new RUPV outflow route to the left atrium (LA) was subsequently constructed through the surgically created ASD. A cavoatrial pathway between the cranial side of the SVC stump and the right atrial appendage (RAA) was reconstructed using a ringed extended polytetrafluoroethylene (EPTFE) prosthesis with a diameter of 16 mm.

**Fig. 3:**
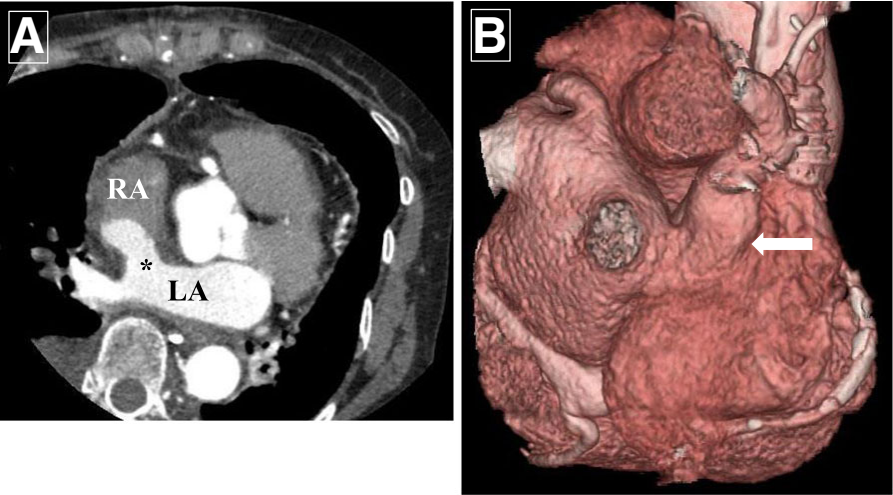
Postoperative computed tomography (CT) findings. **a** Left atrium (LA) and right atrium (RA). *Surgically created atrial septal defect. **b** Three-dimensional CT shows new pathway from right upper pulmonary vein to LA (*arrow*)

The patient tolerated all procedures well and was discharged with normal sinus rhythm after recovery from temporary sinus bradycardia. Anticoagulation therapy with oral warfarin was postoperatively prescribed for 3 months, which is standard procedure after bioprosthetic valve replacement. Postoperative echocardiography showed flow from RUPV to the LA through the new ASD without a significant pressure gradient or congestion. Postoperative contrast-enhanced CT showed that the RUPV flowed into the LA through the new pathway without intracardiovascular thrombus (Fig. 3). The patient remains free of dyspnea, arrhythmia, and thrombotic events, and echocardiography at 4-year follow-up did not reveal evidence of stenosis or obstruction of the reconstructed pathway.

## Discussion

The approximate overall incidence of congenital PAPVR is 0.5% [1]. The most frequent type of PAPVR involves RUPV inflow into the SVC associated with an ASD in about 90% of patients [2]. The incidence of congenital PAPVR involving the right PV with an intact atrial septum is even lower. Isolated PAPVR is initially asymptomatic, but progressive right heart failure or pulmonary hypertension results in the emergence of symptoms [3]. Because PAPVR is usually diagnosed and surgically treated in childhood, few surgical reports have described older adults who have PAPVR, particularly with an intact atrial septum.

The surgical indications and strategy for treating isolated PAPVR remain controversial because of its extremely low incidence. The hemodynamics and clinical features of isolated PAPVR seem to resemble those of ASD, and Majdalany *et al.* [3] stated that regardless of complexity, surgical repair should be considered for isolated PAPVR with signs of RV overload, because their series did not include any surgical mortality and morbidity rates were low. We selected surgical repair for our patient because she was symptomatic and had a dilated RV and high Qp/Qs in addition to the surgical indication for typical ASD.

Surgical repair for PAPVR aims to reestablish systemic and pulmonary circulation, and the strategy will vary depending on the anatomical characteristics of PAPVR. However, the key features of the strategy are as follows. The first is to reconstruct the SVC and PV to create a cavity that is sufficient to avoid stenosis or obstruction. The second is to protect the SN or the SN artery to avoid arrhythmia, particularly SN dysfunction, which sometimes requires permanent pacemaker implantation. The third is to prevent thrombosis associated with prostheses, which perhaps ought to be avoided for infants from the perspective of growth.

The single- or double-patch technique seems effective for low PAPVR when the RUPV interflows into the cardiac side of the SVC. A single patch is simpler and can be a first choice of surgical repair for low PAPVR; however, it could result in stenosis or obstruction of the SVC or PV flow tract for high PAPVR [4]. A double patch could avoid stenosis or obstruction of the reconstructed pathway, but it might be associated with SN dysfunction and PV stenosis [5]. Williams *et al.* [6] and Warden *et al*. [7] originally described caval division in 1984. This procedure aims to maintain a sufficient cavity to avoid stenosis or obstruction of the SVC or PV flow tract and to protect SN function [2]. We applied this technique to our patient because the RUPV interflow to the SVC was higher than at the cavoatrial junction with a large orifice. In addition, a congenital ASD that accompanies PAPVR is allowed to remain open to allow a rerouted pathway. Therefore, a new ASD should be surgically created to repair an isolated PAPVR, and the position and extent of such an ASD should play an important role in the rerouted hemodynamics. We created an ASD in the cranial position from the cranial margin of the fossa ovalis, considering the length of reconstructed pulmonary drain. Suturing the left and right atrial endocardia seemed to help maintain the circular form of the ASD, which postoperative echocardiography showed was free of both congestion and a pressure gradient. Direct suture of the SVC to the RAA or reconstruction with a conduit of autologous pericardial patch or an atrial pedicle flap [6] should be considered, particularly for infants. However, the cranial SVC stump was inadequate to reach the RAA in our patient, and thus a prosthesis was used for reconstruction. In addition, the EPTFE graft provided an antithrombogenic effect. The long-term outcomes of caval division should be good, but although this procedure was safe and effective, it also carried the risk of postoperative PV-LA flow tract stenosis, as well as baffle leakage and obstruction [2, 3]. Careful periodic follow-up is thus needed after these procedures.

## Conclusions

We surgically repaired PAPVR with an intact atrial septum using caval division with pericardial patch baffling, a surgically created ASD, and SVC reconstruction using a prosthetic graft in a symptomatic older adult with an excellent long-term outcome.

## Abbreviations

ASD: Atrial septal defect
CT: Computed tomography
EPTFE: Extended polytetrafluoroethylene
LA: Left atrium
PAPVR: Partial anomalous pulmonary venous return
PV: Pulmonary vein
Qp/Qs: Pulmonary blood flow/systemic blood flow ratio
RA: Right atrium
RAA: Right atrial appendage
RUPV: Right upper pulmonary vein
RV: Right ventricle
SN: Sinus node
SVC: Superior vena cava
